# The crosstalk between lung cancer and the bone marrow niche fuels emergency myelopoiesis

**DOI:** 10.3389/fimmu.2024.1397469

**Published:** 2024-08-01

**Authors:** Evelyn Calderon-Espinosa, Kirsten De Ridder, Thomas Benoot, Yanina Jansen, Domien Vanhonacker, Robbe Heestermans, Ann De Becker, Ivan Van Riet, Lore Decoster, Cleo Goyvaerts

**Affiliations:** ^1^ Laboratory for Molecular and Cellular Therapy (LMCT), Translational Oncology Research Center (TORC), Department of Biomedical Sciences, Vrije Universiteit Brussel, Brussels, Belgium; ^2^ Laboratory for Molecular Imaging and Therapy (MITH), Vrije Universiteit Brussel, Brussels, Belgium; ^3^ Department of Chemistry, University of Warwick, Warwick, United Kingdom; ^4^ Department of Thoracic Surgery, University Hospitals Leuven, Leuven, Belgium; ^5^ Department of Anesthesiology, Perioperative and Pain Medicine, Vrije Universiteit Brussel (VUB), Universitair Ziekenhuis Brussel (UZ Brussel), Brussels, Belgium; ^6^ Department of Hematology, Team Hematology and Immunology (HEIM), Translational Oncology Research Center (TORC), Vrije Universiteit Brussel (VUB), Universitair Ziekenhuis Brussel (UZ Brussel), Brussels, Belgium; ^7^ Department of Medical Oncology, Team Laboratory for Medical and Molecular Oncology (LMMO), Translational Oncology Research Center (TORC), Vrije Universiteit Brussel (VUB), Universitair Ziekenhuis Brussel (UZ Brussel), Brussels, Belgium

**Keywords:** lung cancer, hematopoiesis, immunotherapy, emergency myelopoiesis, bone marrow, biomarkers

## Abstract

Modest response rates to immunotherapy observed in advanced lung cancer patients underscore the need to identify reliable biomarkers and targets, enhancing both treatment decision-making and efficacy. Factors such as PD-L1 expression, tumor mutation burden, and a ‘hot’ tumor microenvironment with heightened effector T cell infiltration have consistently been associated with positive responses. In contrast, the predictive role of the abundantly present tumor-infiltrating myeloid cell (TIMs) fraction remains somewhat uncertain, partly explained by their towering variety in terms of ontogeny, phenotype, location, and function. Nevertheless, numerous preclinical and clinical studies established a clear link between lung cancer progression and alterations in intra- and extramedullary hematopoiesis, leading to emergency myelopoiesis at the expense of megakaryocyte/erythroid and lymphoid differentiation. These observations affirm that a continuous crosstalk between solid cancers such as lung cancer and the bone marrow niche (BMN) must take place. However, the BMN, encompassing hematopoietic stem and progenitor cells, differentiated immune and stromal cells, remains inadequately explored in solid cancer patients. Subsequently, no clear consensus has been reached on the exact breadth of tumor installed hematopoiesis perturbing cues nor their predictive power for immunotherapy. As the current era of single-cell omics is reshaping our understanding of the hematopoietic process and the subcluster landscape of lung TIMs, we aim to present an updated overview of the hierarchical differentiation process of TIMs within the BMN of solid cancer bearing subjects. Our comprehensive overview underscores that lung cancer should be regarded as a systemic disease in which the cues governing the lung tumor-BMN crosstalk might bolster the definition of new biomarkers and druggable targets, potentially mitigating the high attrition rate of leading immunotherapies for NSCLC.

## 1 Introduction

Lung cancer remains the leading cause of cancer related death, reflected by a staggering 1.79 million deaths in 2020 alone (1). Non-small cell lung cancer (NSCLC) accounts for ~85% of all cases and is further subdivided into adenocarcinoma (LUAD), squamous cell carcinoma, and large cell carcinoma. The other 10-15% of lung cancers are represented by fast spreading small cell lung cancer, more slowly growing carcinoid tumors and rare types such as adenoid cystic carcinomas and sarcomas.

Though current lung cancer incidence mortality rates are highest in economically developed countries, they are gradually decreasing, mirroring declines in tobacco smoking next to therapeutic improvements for advanced-stage patients, including targeted and immunotherapies (1). At present, the main biological targets of immunotherapy are immune checkpoints, which aid cancer cells to deceive the patients’ immune system. Via delivery of immune checkpoint inhibitors, mostly monoclonal antibodies targeting the Programmed Death - (Ligand) 1 (PD-(L)1) or Cytotoxic T-Lymphocyte Associated protein-4 (CTLA-4) pathways, antitumor immunity can be unleashed (2). Alas, immune checkpoint blockade (ICB) can coincide with serious immune-related adverse events, objective response rates remain <25% in unselected NSCLC patients and most responders eventually develop progressive disease. These sobering facts reflect the multitude of unaddressed resistance mechanisms to immunotherapy that remain today (3).

Histological expression of PD-L1 and tumor mutational burden (TMB) emerged as predictive biomarkers for response to ICB in NSCLC (4). Unfortunately, their predictive power is often unsatisfactory and doesn’t explain the nature of the resistance hubs that could aid oncologists during (combination) treatment decision making.

In search of additional biomarkers and targetable mechanisms of resistance to ICB, ample studies assigned a leading role to the tumor micro-environment (TME) (5, 6). Cytometry-based compositional analyses studies of the lung TME collectively demonstrated a predominance of T lymphocytes followed by B lymphocytes, neutrophils (Neu), and mononuclear phagocytes (7–11). Most bulk RNA sequencing studies of baseline NSCLC biopsies boil down to the observation that a ‘hot’ TME, characterized by activated effector T cells, interferon γ (IFNγ) signature and/or presence of an antigen presentation machinery, has strong predictive power for response to ICB. Noteworthy, most of these predictive gene signatures depart from a predefined lymphocyte-related gene set (12–15). As a result, most of these studies omit the relevance of the abundantly present Neu and mononuclear phagocytes for response and/or resistance to ICB.

By zooming in on the few studies that used a less or unbiased approach to establish an ICB predictive gene set (16–18), decisive roles for myeloid cell-related signatures have been demonstrated. When Hwang et al. tested the predictive value using a 395-gene panel, significant predictive power could be assigned to a T cell next to a M1 macrophage (Mac) characterizing signature (19). By using virtual microdissection of the bulk transcriptome of the TCGA LUAD cohort at single-cell resolution, Wu et al. defined two myeloid cell infiltration clusters, MSC1 and MSC2, significantly associated with a longer and shorter overall survival (OS) and response to ICB resp. In brief, the MSC2 signature was characterized by LAMP3^+^ dendritic cells (DCs), TIMP1^+^ Macs and S100A8^+^ Neu while the MSC1 profile showed higher levels of CD1c^+^ or CLEC9A^+^ DCs; IFITM2^+^, SELENOP+ or PPARg^+^ Macs and IFITM2^+^ Neu (20, 21). When Salcher et al. focused on the ICB predictive power of Neu subclusters within the NSCLC TME specifically, they defined a 38-gene encompassing tissue-resident Neu signature that was associated with anti-PD-L1 therapy failure in the randomized POPLAR and OAK clinical trials (22). Similarly, Peng et al. recently showed that NSCLC patients with a high TMB but low ‘Neu differentiation expression gene score’ were most likely to benefit from ICB (23).

Hence predictive signatures for ICB efficacy are likely to span more genes than the ones linked to activated lymphocytes and suggest that the lung tumor infiltrating myeloid cells (TIMs) deserve more attention for their biomarker potential to predict both response and resistance to ICB (9, 10, 21, 22, 24–28). Unlike lymphocytes, the proliferative and longevity potential of most myeloid cell subsets is low. This implies that TIM’s ampleness within the lung TME is the result of their continuous recruitment from their main cradle: the bone marrow niche (BMN). Nevertheless, the crosstalk between progressive solid cancers such as NSCLC and the distant BMN remains vastly understudied. This is attributable to the fact that in depth analysis of the BMN is hampered by the marrow’s inaccessible location, low fractions of hematopoietic stem and progenitor cells (HSPCs) (<1%) next to the heterogeneous and continuously differentiating character of hematopoiesis leading to its difficult nature to culture *ex vivo*. Thus, untapped biomarker and target potential might lie in the cues that govern tumor instituted alterations in hematopoiesis and TIM-propagating myelopoiesis in particular.

## 2 Recent insights in hematopoiesis

In adult life, the primary source of myeloid cells is the BMN, where the vital biological process known as hematopoiesis occurs. Hematopoiesis exhibits a continuous hierarchical differentiation process that is tightly regulated by lineage-determining transcription factors (TFs) of which many are highly conserved in mouse and human (29). During embryonal development, intra-aortic cells within the aorta-gonad-mesonephros generate the first multilineage hematopoietic stem cells (HSCs). The latter represent a scarce population of self-renewing cells that translocate to the BMN to constitute most hematopoietic cells during adult life. In addition, HSC-independent hematopoiesis is also initiated during embryogenesis which partly persists in the adult hematopoietic system. HSC-independent lineages are either short-lived, such as primitive erythrocytes, or long-lived, such as tissue-resident Macs (30, 31). Myeloid cells represent a heterogeneous crowd of subsets with embryonic and/or hematopoietic origin (32–34). Fate mapping, transposon, Cre-loxP-mediated barcoding and single cell (sc) RNA-seq studies are continuing to refine our understanding of the compelling hematopoietic trajectories, of which we provide an updated overview below (35, 36).

### 2.1 LSK compartment: HSC and MPPs

**Figure 1:**
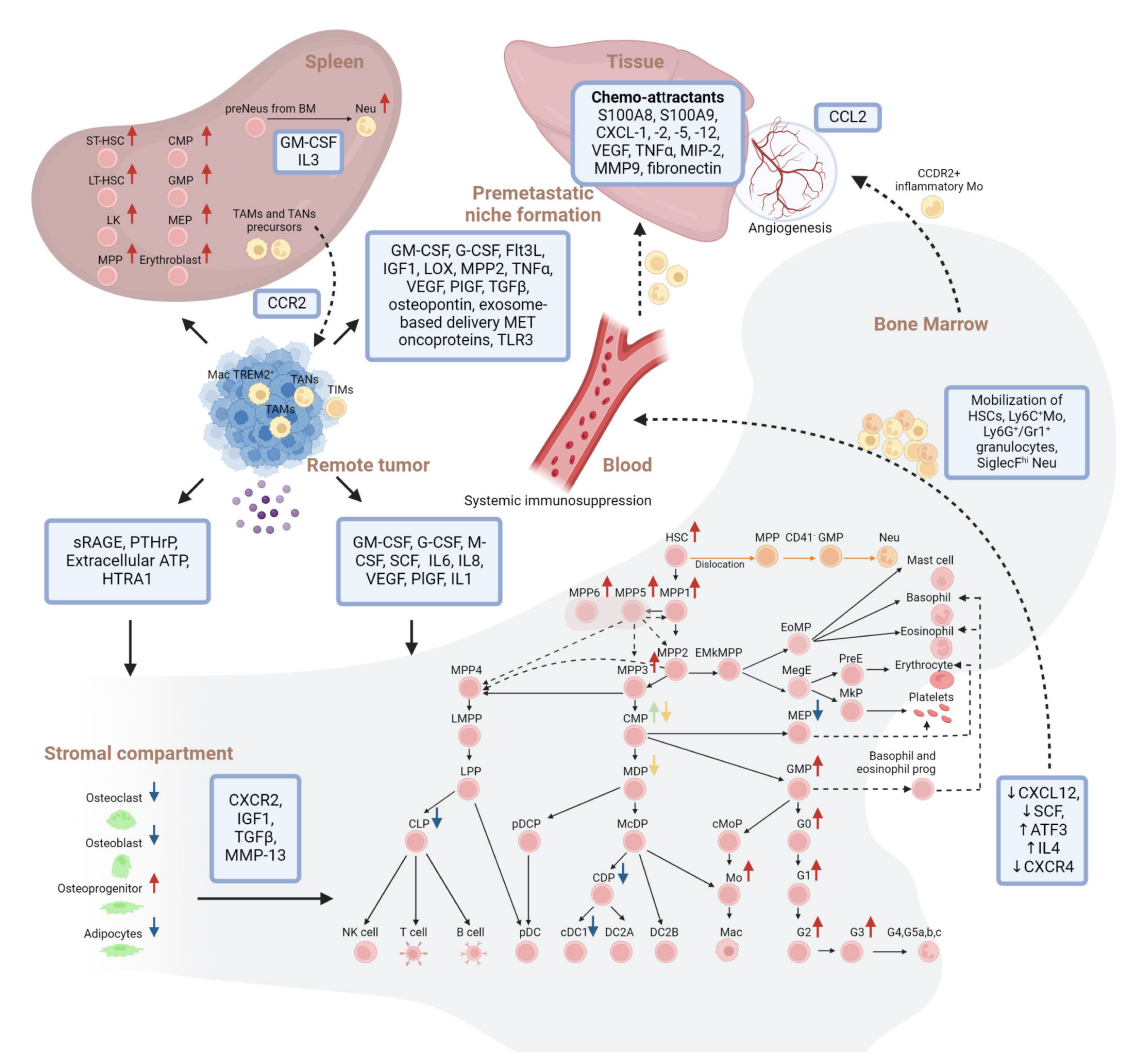
Overview of solid tumor installed cellular and molecular hematopoietic perturbations in human and mouse models. Hierarchical model of continuous hematopoiesis in mouse is shown. Interactions between remote tumor, Bone marrow, stromal compartment, spleen and premetastatic niche formation are indicated. Red arrows indicate increase, blue arrows indicate decrease, green and yellow arrows indicate lack of consensus on changes in the abundance of the respective populations within the bone marrow niche during solid cancer progression. Dashed lines indicate mobilization of cells. Orange lines indicate the alternative trajectory of neutrophils due to the bone marrow-tumor crosstalk. LK, Lin^-^Sca-1^-^c-Kit^+^; HSC, Hematopoietic Stem Cell; MPP 1/2/3/4/5/6, MultiPotent Progenitor 1/2/3/4/5/6; LMPP, Lymphoid-primed MultiPotent Progenitor; EMkMPP, Erythroid-Megakaryocyte-primed MultiPotent Progenitor; EoMP, Eosinophil-basophil-Mast cell Progenitor; MegE, pre-Megakaryocyte-Erythroid progenitor; PreE, Pre-colony-forming-Erythroid progenitor; MkP, Megakaryocyte Progenitor; CMP, Common Myeloid Progenitor; MEP, Megakaryocyte/Erythrocyte Progenitor; LPP, Lymphoid-Primed Progenitor; MDP, Monocyte/Dendritic cell Progenitor; GMP, Granulocyte/Monocyte Progenitor; GP, Granulocyte Progenitor; CLP, Common Lymphoid Progenitor; NK cell, Natural Killer cell; pDCP, plasmacytoid Dendritic Cell Progenitor; McDP, Monocyte/conventional Dendritic cell Progenitor; cMoP, common Monocyte Progenitor; Mo, monocytes; CDP, Common Dendritic cell Progenitor; (c)DC1/2A/2B, (conventional) Dendritic Cell 1/2A/2B; pDC, plasmacytoid Dendritic Cell; G0, granulocyte progenitor; G1/G2/G3, pre/pro/immature neutrophils; G4,G5a,b,c, mature neutrophils; (pre)Neu, (pre)neutrophils; Mac, macrophage; (ST)/(LT)-HSC, Short-term/long-term- HSC; TA(M)/(N), Tumor associated Mac/Neu; TIM, Tumor infiltrated myeloid cells; sRAGE, soluble form of the receptor for advanced glycation end; PTHrP, parathyroid hormone-related protein; ATP, adenosine triphosphate; GM-CSF, granulocyte-macrophage colony-stimulating factor; G-CSF, granulocyte colony-stimulating factor; M-CSF, macrophage colony-stimulating factor; SCF, stem cell factor; IL1/4/6/8: Interleukin 1/4/6/8; VEGF, vascular endothelial growth factor; PIGF, placental growth factor; ATF3, activating transcription factor 3; CXCR2, C-X-C Motif Chemokine Receptor 2; IGF1, insulin-like growth factor 1; TGFβ, transforming growth factor beta; HTRA1, *HtrA* serine peptidase 1; LOX, hypoxia-installed lysyl oxidase. Created with BioRender.com.

The murine Lin^−^Sca-1^+^c-Kit^+^ (LSK) or human CD34^+^ compartment represents the HSCs, including the multipotent progenitors (MPPs). They lodge at the top of the hierarchical ladder and withhold high self-renewing but low proliferative capacity (37). For years, HSCs were subdivided in long-term (LT-HSCs) and short-term HSCs (ST-HSCs) according to their reconstituting potential (38). Yet recent scRNA-seq-based characterization of the LSK compartment with functional reconstitution assays, redistributed this compartment into HSCs and six different types of MPPs (**Figure 1**, **Supplementary Table S1**). Within the latter, erythroid-primed MPP2, myeloid-primed MPP3, and lympho-erythromyeloid-primed MPP4 have been defined (39, 40). Sommerkamp et al. further report on a close relationship between MPP1 and MPP5 placing them downstream of HSCs but upstream of the less potent MPP2/3/4, while MPP6 resides in a cloud with MPP5, be it with higher multilineage potential than the latter. Further, MPP5 seems to act as a reservoir during emergency myelopoiesis (37). These findings suggest that a functional hierarchy, consisting of progenitors at varying degrees of lineage priming is already installed within the multipotent HSC/MPP fraction. Multi- and oligopotency characterizing TFs are *Hlf* and *ESAM* which are downregulated upon specification and commitment resulting in progressive loss of alternative fates along all lineages (35, 39). Additionally, the stem/progenitor state is stabilized by the regulation of the *Gata2*, *Tal1/Scl* and *Fli1* triad (41).

### 2.2 Committed-lineage progenitors

Downstream of the multipotent HSCs and MPPs, the original lineage bifurcation model strictly separated oligopotent Common Lymphoid Progenitors (CLP) from Common Myeloid Progenitors (CMP) which could further diverge into Megakaryocyte/Erythrocyte Progenitors (MEPs), Monocyte (Mo)**/**Dendritic cell (DC) Progenitors (MDPs) and Granulocyte/Mo Progenitors (GMPs). However, this model has been the subject of ongoing revision in the scRNA-seq era. On the one hand, a Lymphoid-primed MultiPotent Progenitor (LMPP) with combined lymphoid and myeloid potential has been shown to gradually lose myeloid potential by upregulating the interleukin-7 receptor α-chain (*IL7Rα*) and differentiate into Dach1^-^ Lymphoid-Primed Progenitors (LPPs) prior to CLPs (29, 33, 35, 42–45). On the other hand, erythroid-versus-myeloid fate decisions seem to be made prior to CMP commitment (36, 40, 46), calling for in-depth revision of all CMP progeny (MEPs, MDPs and GMPs) as well (46). While the Flt3L^+^CD115^lo^ CMPs give rise to oligopotent MDPs, the Flt3^-^CD115^-^ CMPs give rise to GMPs. The former are defined by TF co-expression of *CEBPA*, *IRF8* and *RUNX1* (44). MDPs can give rise to *IRF8/SATB1* plasmacytoid DC (pDC) Progenitors (pDCPs) and *HLA-DR*/*IRF8*/*CEBPA* Mo**/**conventional DC (cDC) Progenitors (McDPs). The latter bifurcate in *CD11c*/*CD172ab*/*CD14* (human) or Ly6C^+^ Mo (mouse) or *IRF8/HLA-DR/CD84/CD172ab* Common DC Progenitors (CDPs) (44). In contrast, GMPs (*Kit*, *CD34*, *Sox4*) are very heterogeneous at the transcriptomic and proteomic level and can adjust their functional output towards common Mo Progenitors (cMoP) and Granulocyte Progenitors (GPs) (32).

### 2.3 DC trajectory

In general, DC progenitors rely on Flt3 and Flt3L for their differentiation and proliferation into pDCs, cDC1 and cDC2. In addition, cDC1 and cDC2 differentiation depends on the specific TF expression of *IRF8*, *BATF3* and *ZFP366* versus *IRF4* and *KLF4* resp. While *XCR1*/*Clec9A*/*CADM1* cDC1s seem to originate solely from CDPs, recent paradigm shifts are sharpening the ontogeny of cDC2s and pDCs. In case of cDC2s, murine T-bet^+^ DC2A and RORγt^+^ DC2B have been described which align with human CD5^+^ DC2s and CD14^+^ DC3s (47). Recently, Liu et al. further demonstrated these DC subsets have a different ontogeny. Whereas ESAM^+^ DC2s are derived from CDPs, the CD172a^+^ CD16/32^+^ DC3 fraction seems to develop directly from Ly6C^hi^ McDPs (48). Further, CD123^+^ pDCs can be derived from MDPs, yet they predominantly originate from IL7Rα^+^ LPPs requiring high expression of *IRF8* and *TCF4* for pDC development and identity resp. Although MDP and LPP derived pDC subsets can secrete IFN type 1, only the former share with cDCs the ability to process and present antigens (49).

### 2.4 Monocyte differentiation

Both in mice and human, Mos are subdivided in *IRF8*-dependent Ly6C^hi^ or CD14^+^CD16^-^ classical and *IRF8*/*Nur77*-dependent Ly6C^-^ or CD14^lo^CD16^+^ non-classical Ly6C^-^ Mo resp. Also, they can emerge from at least two different lineage tracks defined by the complementary oligopotent GMPs and MDPs (32, 34). Interestingly, GMPs produce **“**Neu-like**”** Ly6C^hi^ Mo while MDP-derived Mo show similarities in gene expression with Macs and DCs. Both GMP- and MDP-derived Mo yield Macs but only the MDP pathway gives rise to MoDCs (32, 34). In general, Mos development largely depends on the expression of *Csf1r* (CD115) (48, 50) to result in the expression of Mo characterizing markers such as *Fcer1g*, *F13a1*, *Irf8*, *Klf4*, *Zeb2* and *Irf5* (35, 51). Relative to other myeloid cells, Mos have an exceedingly short transit time through the BMN and are rapidly released into the circulation after their last division, governed by CCR2 and CX3CR1, where the latter receptor prompts survival of Ly6C^lo^ Mo. In contrast, CXCR4-signaling represents an anchoring force that retains Ly6C^hi^ Mos in the BMN, identifying them as transitional pre-Mos that replenish the mature Mo pool for peripheral responses (35, 51). Notably, Mos also contribute to the replenishment of yolk sac-derived tissue-residing Macs in tissue and gender dependent fashion (32).

### 2.5 Granulopoiesis

The neutrophil differentiation trajectory is defined by at least 5 stages (G0-G5) where the earliest GPs (G0), embedded in the GMPs, initiate granulopoiesis through *Runx1*, *Cebpa*, *Gstm1*, *Per3* and *Ets1* expression (35, 52–55). Subsequent differentiation into proNeu (G1), results in full granulocyte commitment and maturation via TF ThPOK (35, 52–55). This is followed by a transcriptional shift to *Gfi1* and *Cebpe* in the preNeu (G2) which represses Mos on MDP fate (*Irf8*, *Klf4* and *Zeb2*). The latter are still proliferative in the BMN and spleen but hardly found in blood due to their poor motility. Further silencing of *Gata1* and *Gata2* results in non-proliferating immature Neu (G3). Finally, *PU.1*, *Cebpd*, *Runx1*, and *Klf6* upregulation drives the generation of mature Neu (G4 mainly in BMN and G5a, b and c mainly in blood) which substitute their proliferative capacity for trafficking and effector functions. In line, they are characterized by Neu lineage characteristic expression of Elastase (*Elane*) and ficollin (*Fcnb*) (53). Notably, the temporal expression of the *Cebp*-family members mirrors the pattern of granule enzyme expression; primary (e.g. *Mpo*), secondary (e.g. *Ltf*) and tertiary granules (e.g. *Mmp8*) are expressed at the G0-G1, G2-G3 and G4-G5 stage, corresponding to *Cebpa*, *Cebpe*, and *Cebpd* expression resp. After maturation, Neus are retained in the BMN through CXCR4 while CXCR2 drives their release into the circulation (56). During inflammation, granulocyte colony-stimulating factor (G-CSF) further potentiates their mobilization.

Recent scRNA-seq studies on murine and human myeloid progenitors suggest that eosinophils, basophils (characterized by high expression of TFs *Gfi1, Il5ra, Prg2, Epx* or *Lmo4, Runx1* resp.) and mast-cells, bifurcate directly from MPPs instead of GMPs. Specifically, *Gata1* expression has been shown to establish an early branch point to separate lineage-commitment. Whereas *Gata1^-^* progenitors commit to the LMPPs and GMPs to hatch lymphocytes and Neu/Mo-restricted lineages resp., *Gata1^+^* progenitors give rise to co-segregated commitment of Eosinophil/basophil/Mast cell Progenitors (EoMP) and a Megakaryocyte/Erythroid lineage (MegE) (57–59). The inflection point at erythroid and megakaryocyte lineage bifurcation is characterized by downregulation of *RUNX1*, *GATA2* and *PBX1* (44).

### 2.6 Stromal involvement in hematopoietic regulation

In the highly specialized BMN, HSCs team up with stromal cells of mesenchymal, endothelial and neuronal origin (60–64). To safeguard the balance between HSC maintenance and output, extrinsic and intrinsic factors are continuously engaged. Extrinsic factors include hypoxia, growth factors (GFs), direct cell-cell contacts, and signaling pathway activating morphogens, next to intrinsic factors encompassing TFs, cell cycle regulators, epigenetic proteins, and miRNAs, as extensively reviewed elsewhere (65). The curated orchestration of hematopoiesis is further reflected in the establishment of bidirectional interactions between HSCs and a variety of endothelial cells and mesenchymal stromal cells (MSCs) in spatially restricted microanatomical niches (64, 66).

The type of blood vessels has been used to subdivide the BMN into a central sinusoidal niche, an arteriolar niche and a peripheral endosteal niche with transition zone vessels. While the former comprises highly permeable vessels to promote HSC proliferation, activation and leukocyte trafficking, the latter two support HSC regeneration and maintenance by keeping them in a quiescent state through reduced reactive oxygen species (ROS) availability (67–69). In addition, the endosteum harbors bone-modulating osteoblasts and osteoclasts, shown to support HSC maintenance and retention amongst others mediated through production of G-CSF, stromal-cell-derived factor 1 (CXCL12 or SDF-1), stem cell factor (SCF) and Notch ligand jagged 1 (JAG1) (70–73).

The vast majority of HSCs reside in the central sinusoidal niche in direct contact with sinusoidal endothelial cells and perivascular mesenchymal progenitors including adipogenic leptin receptor^+^ CXCL12-abundant reticular (CAR) cells (69, 74–80). Via their paracrine secretion of CXCL12 and SCF, the CXCR4 and c-Kit receptor on HSCs are respectively triggered, cardinal for hematopoietic multilineage differentiation (76, 81, 82). Baccin et al. additionally allocated an osterix^+^ osteogenic CAR population to the vicinity of arterioles and non-vascular regions and concluded that the adipogenic- and osteogenic CAR cell populations represent the main producers of key cytokines and GFs (such as CXCL12), suggesting their localization within the sinusoidal and arteriolar niches resp. establishes unique hubs of HSC differentiation and maintenance (61). In addition, Li et al. recently defined 3 stromal niche populations with distinct HSC regulatory impact (64). In addition, they predicted that bidirectional communication enables HSCs and derivates to regulate stromal cells as well. Hence, homeostasis reinstating feedback mechanisms can be activated such as adipogenic CAR cell activation through IL1 secretion of activated platelets (83).

Overall, technological advances are currently sharpening our understanding of the intricate hematopoietic process. Nevertheless, we cannot speak of a complete fathoming of all hematopoietic tributaries yet, calling for continued research of the BMN in healthy and diseased subjects.

## 3 Recent advances in singe cell omics reveal a complex lung TIM crowd

scRNA-seq analysis represents a very powerful tool that recently barged into the onco-immunology field. Through this approach, unbiased and in-depth insights have emerged that foster an unprecedented understanding of biological phenomena on transcriptional single cell level. Especially compared to flow cytometry- and microscopy-based approaches which rely on antibodies to pre-defined markers. In recent years, scRNA-seq of NSCLC biopsies markedly deepened our understanding of the TME in which especially the TIM compartment appeared to hold an underestimated myriad of subsets with distinct progenitors and functions (21–23, 25–27, 84–89). Moreover, TIMs have garnered increasing attention for their potential as biomarkers to predict both response and resistance to ICB. Yet each scRNA-seq study used different tumor samples and sequencing techniques. As a result, a plethora of TIM subsets have been described, yet to our knowledge, no side-by-side overview of the most recent scRNA-seq studies has been generated to define the subsets, perpetuated by most of these studies.

So far, consensus has been reached on at least 5 main ontogenetically discernable human lung TIM populations: DCs, Mos, Macs, Neus and mast cells (**Table 1**). For lung TME-associated DCs, 5 subclusters have consistently been defined: cDC1 and cDC2, mature regulatory DCs (mregDCs, derived from cDC1 and cDC2), pDCs and Mo-derived DCs (Mo-DCs). Lung tumor-associated Mos are repeatedly reported to be of classical or patrolling non-classical origin. In the case of Macs and Neus, much less consensus has been reached across different scRNA-seq studies. We found that for at least 11 Mac and 6 Neu subclusters, two or more independent scRNA-seq studies reported on their presence. The Mac subclusters comprise 3 tissue resident and 8 Mo-derived fractions, while Neus are represented by 2 normal adjacent tissue-associated Neu (NAN) and 4 tumor-associated Neu (TAN) fractions.

**Table 1:** Overview of major lung TIM clusters with respective subclusters defined by scRNA-seq and their respective reported association with response to ICB.

Major cluster	Subcluster	Main characterizing genes		Association with survival and/or response to ICB	Refs.
**DC**	**cDC1**	*XCR1, CLEC9A, CD141, CADM1, IRF8, BATF3, CD226*		/	(21, 22, 25–27, 85, 87–91)
	**cDC2**	*CD1a, CD1c, CD1e, CD207, FCER1A, HLA-DQ, CLEC10A, CLEC4A*		Associated with survival + CD207 is prognostic marker for LUAD, but not for squamous cell carcinoma + increased CX3CL1-CX3CR1 interactions with cancer cells in responders (21, 25, 85, 90)	
	**activated/mregDC**	*LAMP3, CCR7, FSCN1, MARCKSL1, CCL17, CCL19, CCL22, BIRC3, IDO1, CD40, RELB, CD83, CD274, CD200, FAS, ALDH1A2, IL4R, IL4I1, BCL2L1, TNFRSF9*		Associated with lack of response/within LCAM^lo^ profile (21, 26)	
	**pDC**	*GZMB, IRF7, TCF4, LILRA4, TCL1A, PLD4, IRF4/8, CLEC4C, LAMP5, PTPRS, UGCG*		Associated with lack of response (21)	
	**MoDC**	*CCL18, CCL17, CLEC10A, CD163, CD14, MKI67, C1QA/B, S100A8/9*		Associated with survival and response (21)	(25, 26, 87, 88)
**Mo**	**CD14^+^ classical**	*FCN1, VCAN*, *S100A8/9/12, CSF3R AREG, APOBEC3, CD300E, OLR1*		OLR1^+^ Neu-like Mo associated with poor survival (25)	(21, 22, 25, 26, 85, 87, 89–91)
	**CD16^+^ non-classical**	*CD16 (FCGR3A), CDKN1C, LILRB2*, *ITGAL, CX3CR1, SELL, CFP, VNN2*		High CX3CR1^+^ Mo signature is predictive of response (90)
**Macs tissue resident/Alveolar Mac**	**PPARg^+^**	*Marco*	***PPARG, FABP4***, ***MCEMP1*** *, MKI67, STMN1 RBP4, CCL18, CD10, SERPINA1, APOE, CD163, C1QB, CXCL8, IL1B, MSR1, CXCL8, AKR1C3, RSPO3, RND3, HP, FOLR3, GPD1, VSIG4, MRC1, (able to clear surfactant protein)*	Associated with lack of response in LCAM^lo^ profile (26, 89) **<->** Associated with survival and response (21) and elevated after therapy in responders (90)	(21, 22, 25, 26, 87, 89–92)
	**CXCL5^+^**		*NT5E, ANGPTL4, CXCL5, BAG3, MCEMP1, SLAMF9, OLR1*	Associated with worse survival and lack of response (25)	(25, 85)
	**CHI3LA^+^**		*FABP3, FABP5, APOC1, LIPA, MMP7, TIMP3, CHI3L1*	TIMP1^+^ Mac associated with worse survival and lack of response (21)	(21, 25, 85)
**Macs interstitial/Mo-derived**	**SPP1^+^**	*SPP1, TREM2, SLC2A5, CCL7, HAMP*		Associated to superior response in profile at baseline LCAM^hi^ profile at baseline (26) **<->** Increased after therapy in non-responders (via CCL3/4 secretion by TAN-3) (90)	(25, 26, 89, 90, 92)
	**CHIT1^+^**	*CHIT1, GDF15, CTSK*		/	(21, 25)
	**CXCL9^+^**	*CXCL9, CXCL10, CXCL11, GBP1*, ***CCL8*** *, GBP5, SLAMF7, IFITM2*		Associated with worse survival and lack of response (25) **<->** Rich in responders (21)	(21, 25, 85, 90)
	**Cycling/** **dividing**	*TOP2A, MKI67, CDK1, TYMS, CDKN2A, BIRC5, TUBB^+^*		TUBB^+^ associated with shorter overall survival and lack of response (21)	(21, 25, 85, 90, 91)
	**IFN-stimulated**	ISG15, IFIT1, MMP14		/	(85, 89)
	**CD209^+^**	*CD209, CCL13, CCL18, PLTP, F13A1, SELENOP*		Increased after treatment in non-responders (90) **<->** Rich at baseline in responders (21)	(21, 25, 85, 90)
	**C1QA/C^+^**	*APOE, AXL*		/	(26, 89, 90, 93)
	**Other**	***MAF*,** *MERTK, CSF1R, LYVE1 (SEPP1, FCGRT, F13A1, CD163*, ***FOLR2*** *) <-> CX3CR1 (CD74, HLA-DM, HLA-DQ, RGS1, LYZ)*		/	(26, 89)
**Neu**	**NAN-1&2**	*SELL, PTSG2, CXCR2, CXCR1, FCGR3B, MME*	‘classical’ *MMP9, S100A8, S100A9, S100A12*	Poor prognostic factor (21)	(21, 22, 25, 90)
	**NAN-3**		‘IFN-stimulated’ *IFIT1/2/3, ISG15, GBP1/5, RSAD2*, *MX1, IRF7, OAS2*	IFIT1 and ISG15 associated with low survival (25) <-> IFIT3 associated with response (21)	(21–23, 25, 90)
	**TAN-1**	*LOX-1, VEGFA, CD83, ICAM1, CXCR4*	*IL1RN, RIPK271, CD44*	/	(22)
	**TAN-2**		*HLADRA, CD74, HLA-DMB, HLA-DRB1*	/	(22, 23)
	**TAN-3**		*CCL3/4, PI3, C15orf48, CSTB, LGALS3, FNIP2, CSF1*, *IRAK1/2, MIF, CXCL8, CYBB*	Negative association with survival (21, 25)	(21, 22, 25, 90, 91)
	**TAN-4**		‘ribosomal’ *RPS12, RPL3.23, RPN2*	/	(22)
**Mast**		*MS4A2, CPA3, HDC, TPSAB1, TPSB2*		Survival predictors (22, 25, 89)	(25, 89, 91)

In case a subcluster was defined by more than one scRNA-seq study based on overlapping signature genes, we considered this subcluster validated. <->: ‘as opposed to’.

In terms of TIM subcluster specific prognostic and predictive biomarker potential, unanimity also remains scarce. If we only consider the correlations that were validated by more than one scRNA-seq-based study, consensus has been reached for the positive and negative association of cDC2 and mregDCs resp. with response to ICB. In addition, mast cells and TAN-3 have been associated with a better and worse OS, irrespective of the treatment type. Singular studies further ascribe a positive ICB predictive or prognostic role to MoDCs and CX3CR1^hi^ Mo. In contrast, a negative correlation with response to ICB has been reported for pDCs, dividing TUBB^+^ Macs, CXCL5^+^ Macs and CHI3LA^+^ Macs while the presence of NAN 1&2, OLR1^+^ Mo, CXCL5^+^ Macs, dividing TUBB^+^ Macs, TIMP1^+^ Macs and CXCL9^+^ Macs has been associated with worse OS. However, subcluster specific discrepancies have been reported as well. For example, Leader et al. defined the Lung Cancer Activation Module (LCAM) to predict response to anti-PD-(L)1 therapy using scRNA-seq data from lung tumor biopsies at baseline (12). While NSCLC patients with high TMB, mutated *TP53*, and LCAM^hi^ profile with SPP1^+^ Macs (next to PD1^+^ CXCL13^+^ T cells, IgG^+^ plasma cells) showed superior responses, the opposite held true for patients with low TMB, *TP53* wild type and LCAM^lo^ profile characterized by mregDCs and PPARg^+^ Macs (next to naïve and central memory T cells and reduced plasma/B cell ratios). Akin, Wu et al. found that LAMP3^+^ DCs were the only subtype enriched in non-responders, which seems partly in line with the resting mregDC-rich LCAM^lo^ profile. In contrast, both Wu et al. and Hu et al. associated the enrichment of PPARg^+^ Macs (next to NAN-3) with a major pathological response to ICB, while Hu et al. further defined a negative correlation between SPP1^+^ Macs (next to TAN-3) and ICB response. Importantly, Hu et al. analyzed samples after neoadjuvant anti-PD-1 ICB with chemotherapy instead of baseline biopsies (21, 90).

The high variability in subcluster definitions and denoted biomarker potential of lung TIMs can be partly explained by the disparity of the scRNA-seq studies. First, the use of different scRNA-seq platforms has been shown to hamper proper data alignment (22). Second, scRNA-seq studies encompass tumor biopsies from patients with diverse profiles (early versus late stage, histological and genetic subtype) and sampling procedures (intra- versus peritumoral, derived from primary versus metastatic lesion, pre- or posttreatment) (22, 23, 85, 89). Considering that TIM subcluster phenotypes and functions are eminently impressionable by local traits in time and space, evaluation, and comparison of TIM subclusters between different tumor biopsies might be a clinical challenge. To illustrate, both tumor-associated cDC1 and cDC2 have been reported to execute antitumor immune promoting and suppressing activity (88, 94). Within the diversified family of tumor-associated Macs and Neus, this ambiguity seems even more pronounced with assigned functions ranging from pro-angiogenic, pro-inflammatory and immunosuppressive to antigen presenting, CD8^+^ T cell stimulating and tumor eradicating (95, 96).

In conclusion, scRNA-seq delivered an overwhelming amount of new info regarding lung TIM subclusters. However, as the exact TIM clustering method is subject to tumor sample and experimental heterogeneity, their denoted identity and biomarker potential should always be interpreted with caution. Instead of using a snapshot of the TIM composition to predict response to therapy, the question arises whether it is not more interesting to systemically monitor which tumor-derived myelopoiesis perturbing cues are responsible for the influx of TIMs so these can serve as a means of biomarkers and potential targets simultaneously. To illustrate, macrophage-colony stimulating factor (M-CSF or CSF-1) is known to be secreted by tumors to alter remote myelopoiesis. While levels of plasma CSF-1 have been associated with resistance to ICB in NSCLC patients (97), blockade of its receptor CSF1R is currently being investigated to treat advanced malignancies (98).

## 4 Crosstalk between solid cancer and the BMN

The lifespan of the majority of myeloid cell subsets is relatively short. Consequently, the abundance of TIMs within the TME reflects their ongoing recruitment from their primary reservoir: the BMN. Although the communication between solid cancers and the distant BMN remains largely unexplored, the wealth of preclinical and clinical investigations underscore that the progression of solid cancers is a systemic phenomenon, utilizing both intra- and extra-medullary hematopoiesis. Ample preclinical and clinical studies convey that solid cancer progression is a systemic process by exploiting intra- and extra-medullary hematopoiesis. After listing all studies that evaluated solid cancer-related hematopoietic alterations (**Table 2**), it becomes apparent that the latter are most often characterized by emergency myelopoiesis, commonly at the expense of megakaryocyte/erythroid and lymphoid differentiation (111–115, 121, 122, 124–126, 132, 134–137, 139, 140, 144).

**Table 2:** Overview of preclinical and clinical studies which investigated the type and location of hematopoiesis and myelopoiesis perturbing cues in tumor bearing subjects.

Cancer type/model	Main effects on hematopoiesis/ myelopoiesis	Identified myelopoiesis perturbing cues	Refs.
Lewis Lung Carcinoma (LLC) **1992**	LLC progression stimulates myelopoiesis→ °GMP resembling suppressive cells	Low dose IFNγ + TNFα diminishes tumor promoting activities in the BMN and reduces tumor progression	(99)
B6RV2 lymphoma, LLC or Pten^+/-^ tumor bearing Id-mutant (with angiogenic defects) **2001-2006**	Co-mobilization of BM-derived VEGFR2^+^ circulating endothelial precursor cells and pro-angiogenic MMP9-secreting VEGFR1^+^ (myeloid) HSPCs → °rapid tumor neovascularization	- VEGF and PlGF production of tumor cells result in the co-mobilization from BM -Release of SCF results in recruitment of proangiogenic and HSC cells - Perivascular mural cells present CXCL12 to retain CXCR4^+^ endothelial precursors to areas of hypoxia	(100–103)
Intradermal LLC or B16 melanoma with strict vs disseminated metastatic potential resp. **2005**	- BM-derived cell cluster formation at premetastatic sites (lung only for LLC model <-> in multiple tissues for B16 melanoma model) - Main BM-derived cells are VEGFR1^+^/CD133^+^/CD34^+^/c-Kit^+^ HSPCs	Unidentified tumor-secreted factors likely °elevated fibronectin which increases VEGFR1^+^ cell recruitment	(104)
Mouse colon carcinoma or human PC3 prostate adenocarcinoma **2005**	HSCs are distributed in inner tumor mass	Anti-c-Kit neutralizing antibody suppressed tumor angiogenesis	(105)
Intravenous (iv) LLC or B16 10 days after their subcutaneous (sc) implantation **2006**	At premetastatic lung: increased expression of S100A8/9 in CD11b^+^ myeloid cells and endothelial cells of sc LLC-bearing mice	Multiple factors including TNFα, VEGF-A and TGFβ, possibly secreted by primary tumor → °expression of S100A8/9 → °secretion of TNFα and MIP2 and attraction of CD11b^+^ myeloid cells to lung (likely via p38 cascade)	(106)
Anti-VEGF sensitive (TIB6 and B16F1) vs refractory (EL4 and LLC) tumors **2007**	- CD11b^+^/Gr1^+^ myeloid cells install refractoriness to anti-VEGF - Refractory tumors recruit more BM-derived cells to tumors and spleen (at expense of B cells and DCs in BM)	CD11b^+^/Gr1^+^ myeloid cells in refractory tumors showed enhanced 1) angiogenesis (neurotrophin 5, Endo-Lip, angiopoietin-like 6, semaphorin VIb, Eph RA7, Eph RB2 and FGF1 <-> VEGFR-1 does not play a significant role), 2) BMN cell mobilization (G-CSF and MCP-1), 3) inflammation (MIP-2 and IL1) and 4) differentiation/activation of myeloid cells: IL4R, IL13R, TLR-1R and GM-CSF	(107)
FVBN202 transgenic mouse model of spontaneous breast carcinoma (BC) **2009**	- Spleen, blood and BM: peripheral CD11b^+^Gr1^+^ cell expansion	**GM-CSF** (not VEGF nor MCP-1) = main tumor-derived soluble determinant of Gr-1^+^ CD11b^+^ cell generation and maintenance from GMPs (yet *GM-CSF blockade did not completely abrogate this process)*	(108)
Sc 4T1 BC **2009**	Preferential expansion of GMPs and CD11b^+^/Gr-1^lo^ cells in BMN and CD11b^+^/Gr-1^int/lo^ in spleen + ° tumor-induced tolerance	GM-CSF necessary to °preferential expansion and tumor-induced tolerance <-> G-CSF expand preferentially CD11b^+^/Gr-1^high^ cells without °tolerogenic environment	(109)
Ot MDA-MB-231 human BC in nude mice or 4T1 in BALB/c **2009**	- CD11b^+^ cells adhere at premetastatic sites when extra cellular matrix is crosslinked by LOX → respond with increased MMP-2 → °invasion of CD11b^+^ myeloid cells and c-Kit^+^ HSPCs	Hypoxia increases expression of LOX in tumor cells → accumulates at pre-metastatic sites → crosslinks collagen IV in basement membrane → °increases MMP2 which makes sites more permissive for c-Kit^+^ HSC and CD11b^+^ myeloid cell invasion	(110)
Human BPLER or MDA-MB-231 BC (instigators) + weakly tumorigenic HMLER-HR (responders) sc into contralateral flanks **2011**	Sca1^+^/c-Kit^-^ BMN cells (of which 95% CD45^+^) most abundant BM-derived cell fraction in responding tumors, stimulated by instigating tumors - characterized by upregulation of: *GRN, GATA6, RIKEN, Acot4, Krft84, Bat1a, Fcgr1, Defa4, Steap3 and EGFR* <-> within BMN: # of LSK cells reduced	Instigating tumors secrete osteopontin → °granulin producers derived from Sca1^+^/c-Kit^-^ BMN cells (NOT the Sca1^-^ nor Sca^+^c-Kit^+^ fraction) → migrate to responding tumors → °α-SMA^+^ tumor promoting myofibroblasts at premetastatic niche support tumor cell engraftment	(111, 112)
sc LL2 **2011**	- Increase in CD45^+^ LSK HSPCs in tumor over time - Tumor, BMN and blood: HSCs and myeloid populations increased <-> lymphoid and erythroid populations decreased	IGF signaling via IGF1 receptor on HSCs supports tumor outgrowth (primary and metastatic)	(113)
B16F10 vs B16F1 melanoma model + patients **2012**	- Melanoma-specific “exosome signature” characterized by TYRP2, VLA-4, HSP70) and MET - Exosome treatment → °two-fold increase in vasculogenic c-Kit^+^Tie2^+^ progenitors in BMN (<-> other HSPCs **not** affected) - Treatment with B16-F10, but not B16-F1, exosomes increased Met in vasculogenic (Tie2+) and LSK HSPCs in circulation but not in the BMN - CD45^−^c-Kit^low/+^Tie2^+^ circulating HSPCs in metastatic melanoma patients express high MET receptor	- Ras-related (Rab) protein expression associates with melanoma exosome production - MET oncoprotein within exosomes likely enhances BMN cell mobilization	(114)
Ot primary ductal epithelial LSL-Kras^G12D^ cells **2012**	Accumulation of CD11b^+^/Gr1^+^ cells in neoplastic pancreata	Oncogenic Kras^G12D^ enhances production of GM-CSF in pancreatic ductal cells, crucial for accumulation of tumor infiltrating CD11b^+^/Gr1^+^ cells	(115)
KPC pancreatic ductal adenocarcinoma model **2012**	Lin^-^/c-Kit^+^ splenocytes represent highly proliferative precursors of CD11b^+^/Gr1^+^ cells (which was much less pronounced in BM)	**GM-CSF** (tumor or stroma derived) was necessary and sufficient to drive extramedullary HSPC proliferation and development of T-cell suppressing CD11b^+^/Gr1^+^ cells <-> minor impact of other tumor derived factors such as **CXCL1**, CCL2, VEGF, G-CSF or M-CSF)	(116)
Spontaneous ot KP model **2012**-**2013**	Local accumulation of extramedullary HSPCs (mainly GMPs) within splenic red pulp → °generation of TAM and TAN precursors	- Recruitment to spleen requires CCR2 - Overproduction of angiotensin II binds to AGTR1A → °suppression of S1P1 signaling → amplifies splenic (but not BM) HSCs and Ly6C^+^ Mo	(117, 118)
EL-4 thymoma, LLC, 4T1 BC, K-ras/CC10-cre mice, B16F10 melanoma, CT26 colon carcinoma and OP9 stromal cells **2013-2019**	Enhanced Rb-1^low^ ‘Mo-like precursor of granulocytes’ fraction in spleen and BMN with CD11b^+^/Gr1^+^ cell differentiation potential	Expansion was controlled by down-regulation of Rb1 (but not IRF8)	(119, 120)
Different BC lines (MMTVneuOTI/OTII, CMT-93 and 4T1) + B16F1 injected sc or in mammary fat pad (ot) **2013**	- Blood: 7-10-fold increase in granulocytes <-> decrease in erythrocytes and platelets - Spleen: increased cellularity through increase of ST-HSCs, LT-HSCs, LKs, MPPs, MEPs, erythroblasts, Mo and granulocytes - BM: hypocellular with fewer Mo, erythroid cells and megakaryocytes <-> modest increase in granulocytes, increase in ST-HSCs and MPPs <-> fewer/similar LT-HSCs and LKs and no CMP nor GMP changes → **independent of injection site for BC but size-matched B16 melanoma tumors did not induce similar perturbations**	- *In vitro*: BC cell-derived G-CSF can synergize with FLT3L and GM-CSF to expand myeloid progenitors and progeny (but NOT for B16 nor CMT96 colon carcinoma lines) - °histone methylation changes within HSCs in the BMN of tumor bearing mice correlated with gene dysregulation of the Hox family and PRC2 chromatin-remodeling complex (diminished Ezh2 expression, reduced H3K27me3, and Hoxa9 upregulation)	(121)
133 solid cancer patients **2014**	- Blood: decrease in CMPs <-> no differences in MEPs nor CLPs <-> increase in HSCs and MPPs and especially GMPs (4-7 fold) → only CD15^+^/CD133^int/+^ granulocytic precursors and subsets up-regulated <-> declined lymphoid potential) + association between circulating GMP levels and disease progression - Colorectal tumor tissue: increase in CD133^+^ HSCs that co-express myeloid markers CD15 or CD14 + were mainly positive for CXCR4^+^	- GM-CSF increased frequency of GMPs - G-CSF alone did not affect GMP frequency, but had most potent impact on induction of CD14^+^ Mo and CD15^+^ granulocytes + on their expression of IL4Rα and M-CSFR - IL6 alone had only a marginal effect but an additive effect in combination with GM-CSF and G-CSF.	(122)
BC-bearing KEP mice **2015**	- °systemic expansion and polarization of Neu - absence of γδ T cells or Neu profoundly reduces pulmonary and lymph node metastases without influencing primary tumor progression	IL1β elicits IL17 expression from γδ T cells → °systemic, G-CSF-dependent expansion and polarization of Neu	(123)
Transgenic PyMT BC mouse model **2015**	- Lung, blood and spleen: CD11b^+^/Ly6G^+^ Neu expansion <-> no accumulation in TME - BM: twofold expansion of LSK fraction with an increased HSCs (8wks), MPP (at 10wks) and GMPs while *no significant change in CMPs* + decreased erythropoiesis resulting in anemia <-> unlike BM, majority of spleen-residing myeloid precursors were MEPs	Tumor-derived G-CSF → inhibits acquisition of Rb1 expression in Neu (which they normally acquire after they leave the BM) → °ROS producing Rb1^low^, T cell suppressive Neu → enhances Ly6C^hi^, MPP and HSC numbers in the BMN of tumor-bearing mice	(124)
Sc LLC **2016**	- BMN and blood: expansion of HSCs, MPP and GMPs but not CMPs	Immunosuppressive character of GMPs is NO-dependent	(125)
Ot E0771 BC model or M3-9-M rhabdomyosarcoma or iv for °metastases **2016**	- BMN and blood: elevation of LSK HSPCs - ST-HSCs and MPP contributed to elevated expansion of LSK HSPCs in BMN yet LT-HSC numbers remained unchanged - In premetastatic lung: twice as many donor-derived LSK HSPCs developed into CD11b^+^ cells (with more CD11b^+^Ly6G^+^ and CD11b^+^Ly6C^hi^ cells, yet not TAMs)	- FLT3 inhibition diminished both LSK HSPC mobilization and CD11b^+^Ly6G^+^ accumulation at early metastatic sites - Similar to G-CSF, CXCR4 receptor antagonist AMD3100, mobilizes HSPCs + more metastases (not in SCID mice) but did not promote myeloid lineage skewing	(126)
Spontaneous ot KP model, KP1.9 and iv LLC **2017-2020**	- Tumor infiltration of SiglecF^high^ Neu subset with discrete cancer-promoting properties (e.g. via VEGF and ROS) - Higher trabecular bone density in thoracic vertebrae of NSCLC patients <-> controls	Cancer induced sRAGE stimulates bone-resident osteocalcin-expressing osteoblastic cell activity and increases CXCR2 expression → ° Neu mobilization and supply of SiglecF^high^ Neu	(127, 128)
RET melanoma, KPC pancreatic cancer, and TRAMP prostate cancer models next to sc EL4 lymphoma, LLC and CT26 colon carcinoma (sc or iv for LL2) **2018**	- At early cancer stage: enhanced spontaneous migration of BMN Neu with enhanced oxidative phosphorylation and glycolysis but not immunosuppressive - At late cancer stage: immunosuppressive Neu with similar pattern of migration activity as Neu from tumor free mice	Spontaneous migration of BMN Neu is mediated by autocrine ATP signaling	(129)
Ot FC1242 pancreatic tumor model **2018**	- preNeus expand in BMN and spleen under tumoral stress → support recruitment of Ly6G^lo/+^/CXCR2^-^/CD101^-^ immature and Ly6G^+^ CXCR2^+^ CD101^+^ mature Neu to blood and TME - Peripheral abundance of immNeu is most prominent when high tumor burden	/	(53)
BC and pancreatic ductal adenocarcinoma (PDAC) patients + 3 genetic and 4 syngeneic ot mouse models of BC and PDAC **2018**	- patient BM: decreased # of CDPs, pre-DCs, cDC1s and cDC2s <-> expansion of BMN immature granulocytes <-> **MDPs reduced in BC but not PDAC patients** - mouse BM: similar except for inconsistent decrease in cDC2s (unlike cDC1s) **-** patient blood: also reduction in pre-DC <-> increase in immature granulocytes - impaired priming of CD8^+^ T cells and correlation with poor patient outcomes	- tumor-induced GCSF is both necessary and sufficient to reduce cDC1 differentiation by downregulating IRF8 in cDC progenitors (MDPs, CDPs and pre-DCs) directly via STAT3 activation <-> IL6 neutralization reduced immature granulocytes and inflammatory Mo yet not reversed tumor-induced reductions in pre-DCs or cDC1s in BM	(130)
Melanoma patients and murine B16F10 melanoma model **2018**	Increase in very early stage committed unipotent Neu progenitors in human and murine BMN and blood + suppress T cell activation and promotes tumor growth	/	(131)
Transplanted (sc: MC57 fibrosarcoma, MC38 colon carcinoma, B16-F10 melanoma, and 3LL lung tumors), MCA induced (sc), and °spontaneous ot KP model **2019**	In MC57 fibrosarcoma and 3LL implanted, MCA induced and KP model: °emergency myelopoiesis with more proliferative HSCs, MPPs (2 and 3), CMPs and GMPs in BMN (while CLPs remained same) + increased # of HSPCs in spleen and blood + detected in tumor stroma + <-> in MC38 colon carcinoma and B16F10 melanoma: no significant alterations BMN HSPCs	Mainly CD4^+^ T cells within tumor secrete TNFα → activates HSPCs and myeloid differentiation (GM-CSF was only found in conditioned medium of MC38 who had NO significant impact on BM)	(132)
Melanoma and NSCLC patients **2020**	Co-expression of CD71 and CD117 identifies early proliferating unipotent Neu progenitor population in human BM, blood and tumors	/	(133)
Colorectal, glioblastoma, pancreatic, melanoma and 3 BC models (4T1, AT3 and MMTV-PyMT) **2020**	- Spleen, blood and BMN: Ly6G^+^ Neu expansion <-> no accumulation within lymph node nor TME - BMN and TME: similar perturbations in T cell pool next to less mature CD11b^+^ myeloid cells <-> blood and spleen: T cell pool dominated by CD4^+^ T cells	Critical mediator of tumor-driven systemic immune remodeling is IL1a → °increase in G-CSF	(134)
LLC (sc), B16-F10 melanoma (sc or iv) and MC38 (sc) + cancer patients **2018-2020**	- MEP → °CD45^+^ erythroid progenitor cells in spleen and BMN which don’t differentiate to erythrocytes but de-differentiate into multipotent state termed erythroid differentiated myeloid cells with myeloid lineage markers IRF8 and PU.1 and silencing of erythroid lineage markers Gata1 and Klf1 → ° anemia, T cell exhaustion and resistance to anti-PD-(L)1 ICB	- Tumor-derived GM-CSF dedifferentiates CD45^+^ erythroid progenitor cells into erythroid differentiated myeloid cells - Erythroid progenitor cells mediate immunosuppression through ROS production	(135, 136)
sc LLC **2021**	- Blood: °anemia <-> increase in leukocytes - BMN: increase in immature erythroblasts and % of myeloid cells <-> reduced differentiation of MPPs → proportion of LT-HSC, ST-HSC, and MPP stayed unchanged yet CMP/MPP and CLP/MPP ratios decreased - BMN stroma: 1) bone volume/total volume, trabecular number, and cortical wall thickness decreased, 2) osteoclast # increased next to expression of osteoblastic (*Runx2*, *Alpl*) and osteoclastic genes (*Ctsk* and *Acp5*), 3) reduced growth rate of MSCs with elevated osteogenic but reduced adipogenic potential, CXCL12 and SCF expression	Excessive TGFβ deteriorates BMN → °increased phosphorylation of p-Smad2/3, perturbed hematopoiesis and fibrosis with elevated expression of *Acta2* (indicating expansion of myofibroblasts) and the fibrotic genes *Col3a1* and fibronectin	(137)
Different BC models (hu MCF7, hu MDA-MB-231 and mu AT-3) in nude and C57Bl/6 resp. **2021**	°osteoclast maturation and acceleration of bone resorption → release of GFs (e.g., IGF1) which reciprocally stimulate tumor growth and metastasis within the bone	Cancer cells secrete molecules such as PTHrP, which act on osteoblasts → modulate RANKL and OPG expression → °osteoclast maturation Enhanced EZH2 activity → °epigenetic reprogramming of tumor cells in bone microenvironment for further metastases	(138)
MMTV-NeuT spontaneous or transplanted BC models **2020-2023**	Transcriptional modifications in BMN occur at preinvasive stage of disease: - downregulation of adaptive immunity and extracellular matrix proteins - induction of innate immunity and response to danger signals triggered by ATF3 - BMN stromal architecture modification via relocalization and increased density of Nestin^+^/CXCL12^+^ MSCs and CXCR4^+^ myeloid cells	ATP release from BC → °enhanced activation of NLRP3/inflammasome and release of IL1B by BM-MSCs → upregulation and nuclear translocation of ATF3 in BMN → °increased density of Nestin^+^ CXCL12 secreting MSCs and myeloid populations at expense of erythroid and B cells At early stage: ATF3 in HSCs → promotion of CMP/GMP cluster formation <-> At later stage: expansion and release of CXCR4^+^ ATF3^+^ CD14^+^ Mo-myeloid cells that differentiate into Macs in periphery - Deregulation of circulating miRNAs were predicted regulators of downregulated transcripts in BM	(139, 140)
Different BC models: non-metastatic MMTV-PyMT and ot Py230 in C57BL/6 and BALB/C MMTV-Neu model **2023**	BMN: increased # of LT-HSC with increased myeloid differentiation potential → °increase in blood myeloid cells which correlated to tumor burden + altered vasculature in endosteal niche + increased # of MSCs with higher osteoblast (and reduced adipocyte) differentiation potential with increased proportion of LT-HSC near MSCs	Serum: NO increase in G-CSF or GM-CSF yet increase in CD14, MMP3 and MMP9, Angiopoietin-2, WNT1-inducible-signalling pathway protein 1 and the RANKL decoy receptor OPG - MSCs differentially expressed hematopoiesis regulating genes like *Vcam1, Cxcl12, IL7, IL6* and *Csf1*	(141)
ot triple-negative BC PyMT-N, 2208L and 4T1 = Neu-enriched subtype <-> PyMT-M, E0771, T11 and 67NR = Mac-enriched subtype (MES) + sc LLC model **2023**	BMN of Neu-enriched subtype TNBC and LLC: - LT-HSCs, ST-HSCs, MMPs and GMPs increased <-> CMPs, CLPs and MEPs decreased/remained unchanged + enrichment of CD41^-^ GMP clusters near sinusoidal and arteriolar vessels and endosteum - osteoprogenitors (OPs) increase <-> adipogenic differentiation genes reduced in MSCs and OPs - Crosstalk between OPs and GMPs → °systemic accumulation of myeloid cells/protumorigenic Neu BMN and blood: accumulation of myeloid subsets, especially Neu **<-> MES tumors caused no or little changes in myelopoiesis** - TNBC patients: increased HSC/MPPs, GMPs, and Neu	HTRA1 on tumor-derived extracellular vesicles inhibits BMP4 → °upregulates MMP13 in Osterix^+^ Ops → increases CD41- GMP via downregulation of *IL34* and Csf1 likely reduced monocytic-myeloid differentiation	(142)
ot KP lung tumors (upon iv injection) in wt or specific IL4ra ko mice in GMPs (IL4ra^ΔMs4a3^) **2023**	In IL4ra^ΔMs4a3^-cre mice (vs wt) tumor bearing mice: - 85% reduction in tumor burden IL4ra^ΔMs4a3^-cre mice <-> no difference when IL4ra ko in DC, Mo, Neu, Mac or T cells - °inflamed antitumor lung TME state - reduced GMP expansion - Mo expressed higher vs lower levels of genes associated with maturation vs early stages of myeloid development	- BMN basophils are dominant IL4 source → - basophil depletion reduces immune-suppressive myelopoiesis + tumor burden - 8 tumor-derived cytokines synergistically result in IL4 upregulation in basophils: IL18, VEGF-A, IL6, IL1α, IL7, CCL3, IL15 and CSF2	(143)

<->: ‘as opposed to’. °: ‘generation of’.

### 4.1 Emergency myelopoiesis

Paracrine signals from the tumor most often instigate emergency myelopoiesis, entailing activation, expansion and/or mobilization of HSPCs and immunosuppressive myeloid progenitors. In brief, most studies report on a drastic elevation of cells in the LSK compartment (HSCs and MPPs, mainly MPP3), GMPs and early stage committed Neu progenitors in the BMN (**Figure 1**), blood and even in the TME (53, 99, 109, 113, 119, 121, 122, 124–126, 129, 131–134, 141, 142, 145). Although immunosuppressive Ly6C^+^ Mo and Ly6G/Gr1^+^ granulocytic myeloid cells, a.k.a. mo- and g-myeloid-derived suppressor cells resp., have been shown to dominate peripheral blood and tumors (126, 146),, trends for BMN-residing CMPs are less clearly defined than for GMPs. For example, Wu et al. describes a marked decrease of CMPs in blood of 133 solid cancer patients (in contrast to significant increase in HSCs, MPPs and GMPs) (122). Akin, higher frequencies of GMPs but not CMPs have been reported in murine breast and lung cancer models (124, 125, 137, 142). In contrast, Al Sayed et al. showed in fibrosarcoma and different lung models that next to HSCs, MPPs and GMPs also CMPs increased due to their higher proliferative capacity within the BMN (132). In addition, Meyer et al. observed that the increase in immature granulocytes was at the expense of CDPs, pre-DCs and especially cDC1s (130).

### 4.2 Secreted myelopoiesis perturbing cues

Cancer-forced emergency myelopoiesis is orchestrated by tumor secreted myeloid-lineage-specific GFs and cytokines such as granulocyte-macrophage colony-stimulating factor (GM-CSF), G-CSF, M-CSF, SCF, IL6, IL8, vascular endothelial growth factor (VEGF), Placental growth factor (PlGF) and IL1 (100, 101, 108, 109, 115, 116, 121, 122, 147). GM-CSF is by far the most documented tumor-derived hematopoietic perturbator with reported role in amongst others, the expansion of GMPs and immunosuppressive Ly6C^+^ Mo and Ly6G^+^/Gr1^+^ granulocytes in BM, blood and spleen. Moreover, it has been linked to the de-differentiation of erythroid progenitors to multipotent immunosuppressive myeloid progenitors leading to anemia. Notably, anemia is shown to be strongly associated with poor prognosis in most cancer types as well as a compelling risk factor that can obstruct ICB therapy efficacy (122, 124, 135–137). Less consensus has been reached on the role of G-CSF with reports ranging from ‘essential for suppressive Neu expansion and polarization’ at the expense of cDC1 generation (123, 124, 130) over ‘minor impact on HSPC and GMP proliferation and/or immunosuppressive myeloid cell skewing’ (109, 116, 122, 126). Notably several studies reported on the lack of hematopoietic perturbation similarity between different tumor models (121, 130, 142), calling for careful interpretation of studies using different mouse models in terms of tumor type, stage, site and mode of engraftment.

### 4.3 Stromal involvement in hematopoietic regulation during cancer

Interestingly, recent studies on how tumor secreted factors are molding hematopoiesis exactly, are revealing a key role for BMN-residing stromal and differentiated immune cells. Akin, cancer-related bone loss as a result of deteriorated osteoclast and osteoblast numbers and activity has been reported (127, 128, 137, 138). It seems that this mainly involves the direct crosstalk between tumor cells and MSCs, which are skewed towards osteoprogenitors at the expense of adipogenic differentiation (141, 142). Mechanistically, spontaneous orthotopic lung cancer models have been shown to secrete a soluble form of the receptor for advanced glycation end products (sRAGE) which can provoke osteocalcin^+^ osteoblast activity with enhanced CXCR2 expression and subsequent mobilization of tumor-infiltrating SiglecF^hi^ Neu (127, 128). Zhang et al. further describe the involvement of tumor-derived parathyroid hormone-related protein (PTHrP) which bolsters osteoblast maturation by modulating the expression of *RANKL* and its decoy receptor osteoprotegerin (OPG) (138). Osteoclast activity can subsequently release niche and hematopoiesis perturbing factors such as insulin-like growth factor 1 (IGF1) and TGFβ (137, 138). In addition, breast cancer-derived ATP has been shown to activate the NLRP3/inflammasome and release IL1β by BMN-residing Nestin1^+^ CXCL12 secreting MSCs which results in decreased CXCL12 and SCF expression to increase HSC mobilization, ATF3 promoting CMP/GMP cluster formation and expansion of CD14^+^ Mo that differentiate to tumor-associated macrophages (TAMs) (139, 140). Finally, HtrA serine peptidase 1 (HTRA1) on tumor-derived vesicles has been reported to upregulate MMP13 in osteoprogenitors which subsequently induce CD41^-^ GMP clustering and systemic accumulation of protumoral Neu (142).

Aside from stromal cells, also BMN-residing basophils were recently shown to fuel emergency myelopoiesis via their production of IL4 during orthotopic lung tumor progression, resulting in GMP expansion and subsequent increase in the number of lung TIMs such as TREM2^+^ Macs (143). From these relatively disparate findings, it appears that further research is needed to ascertain the exact tumor-specific direct and indirect crosstalk with the different members of the BMN to install tumor promoting emergency hematopoiesis.

### 4.4 Extra-medullary alterations in hematopoiesis

Next to intra- also extra-medullary alterations in hematopoiesis have been reported (108, 119, 120, 124, 132, 134–136). Bayne et al. used the pancreatic adenocarcinoma model KPC to show that Lin^-^/c-Kit^+^ HSCs within the spleen were highly proliferative precursors of Neu and that this phenomenon was much less pronounced in the BMN (116). In line, Sio et al. report on increased splenic but not BMN cellularity with increase in ST-HSCs, LT-HSCs, LKs (Lin^-^Sca-1^-^c-Kit^+^), MPPs, CMPs, GMPs, MEPs and erythroblasts. In contrast, the BMN only revealed an increase in ST-HSCs, MPPs and GMPs whereas the number of LT-HSCs, LKs, MEPs and CMPs decreased (121). In contrast, Casbon et al. found in the PyMT transgenic breast carcinoma (BC) model that the BMN was the main site of Ly6G^+^ cell generation, in accordance with increased frequencies of marrow but not splenic HSCs, MPPs and GMPs (124). Unlike BM, the majority (80–90%) of spleen-residing myeloid progenitors were MEPs, which is in line with later studies on the generation of erythroid differentiated myeloid cells in tumor bearing subjects (135, 136). As such the splenic red pulp acts as an extra reservoir of immunosuppressive TAM and TAN precursors (recruitment to tumor via CCR2) that promote solid tumor progression and metastasis (117). Mechanistically, overproduction of the peptide hormone angiotensin II in tumor bearing mice triggers S1P1 signaling in HSCs and as such amplifies splenic but not BM-derived HSCs which acts upstream of a potent Mac amplification program (118). These findings indicate HSPC mobilization from the BMN to the spleen as well as extramedullary HSPC proliferation (107, 116). For emergency granulopoiesis specifically, preNeus have not been found in blood, ruling out their mobilization from the BMN to spleen making their splenic expansion likely attributable to the production of GM-CSF and IL3 in the spleen microenvironment (53, 148).

### 4.5 Premetastatic niche formation

In addition, systemic tumor-HSPC crosstalk represents a cornerstone for tissue specific premetastatic niche formation. On the one hand systemic release of tumor-derived factors can downregulate CXCR4 resulting in the proliferation and mobilization of HSPCs like VEGFR1^+^ HSPCs, myeloid cell skewed LSKs and α-SMA^+^ cancer-associated fibroblasts activating Sca-1^+^c-Kit^-^ cells to the premetastatic niche (104, 111–113). Primary tumor-derived factors reported to be involved in these processes are GM-CSF, G-CSF, Flt3L, IGF1, hypoxia-installed lysyl oxidase (LOX), MMP2, tumor necrosis factor alpha (TNFα), VEGF, PlGF, TGFβ and osteopontin (5, 100–103, 106, 110–113, 121, 126, 132, 137, 138) as well as exosome-based delivery of MET oncoproteins and TLR3 triggering small nuclear RNAs (114, 149). These factors support recruitment of HSPCs next to Ly6G^+^ Neu and Ly6C Mo myeloid cell skewing at the premetastatic niche which further involves the expression of chemo-attractants like S100A8 and S100A9, CXCL-1, -2, -5 and -12 next to VEGF, TNFα, MIP-2, MMP9, fibronectin, largely dictated by the cellular composition of the TME (25, 106, 149, 150). On the other hand, the BMN is also actively involved in the provision of angiogenic cells such as BM-derived VEGFR2^+^ endothelial progenitor cells and vasculogenic c-Kit^+^Tie2^+^ progenitor cells that contribute to neovascularization of premetastatic sites (101, 151). As such, solid cancer - BMN crosstalk not only impacts hematopoiesis but also tumor vascularization and even angiogenesis as CCL2 gradients recruit CCR2^+^ inflammatory Mos, which produces VEGF to increase vascular permeability (107, 128). This is further strengthened by the observation that anti-c-Kit neutralizing antibody treatment suppressed tumor angiogenesis in a murine colon and human prostate model (105).

Overall, the distant solid tumor – BMN crosstalk can result in a plethora of hematopoiesis perturbing cues which ultimately result in the lamentable characteristics of tumor progression ranging from metastasis over bone loss and anemia. Although this crosstalk may thus be the ideal target to nip the progression of solid tumors in the bud, the exact signals, and cellular actors of this crosstalk are just begun to be uncovered. Our overview further shows that so far both similar and different observations have been made, based on the experimental setup and tumor type, ratifying a great deal of research remains to be done.

## 5 Clinical targeting of myelopoiesis perturbing cues

From the extensive list of identified solid cancer installed myelopoiesis perturbing cues (**Table 2**), only a handful have so far been clinically targeted in NSCLC patients. These include IL6 with Tocilizumab (152–154); CCL2 with Carlumab (155), IL1β with Canakinumab (156–162), VEGF-A with Bevacizumab (163) or Ramucirumab (anti-VEGFR2 (164)), CSF1R with Cabiralizumab (165) or Pexidartinib/PLX3397 (166), IGF1R with Figitumumab (167) and IL4 with Dupilumab (168). While Bevacizumab and Ramucirumab are FDA approved to treat advanced NSCLC in combination with chemo or targeted therapy based on their significant improvement in progression free survival and OS [161, 162], all other agents are still under clinical evaluation. This as monotherapy (Carlumab and Canakinumab) or in combination with chemotherapy (Canakinumab and Figitumumab) or ICB (Tocilizumab, Canakinumab, Cabiralizumab, Pexidaetinib and Dupilumab). As these are mainly represented by phase I/II clinical trials probing drug safety and tolerability, efficacy data are scarce (152–154, 156, 157, 159, 162, 165, 167). However, in case of anti-IL1β therapy with Canakinumab, at least three trials demonstrated a lack of efficacy when delivered as mono- or in combination with chemotherapy (158, 160, 161). Similarly, the phase I/IIa clinical trial in which the safety and ORR were evaluated in solid cancer patients upon combined CSF1R inhibitor Pexidaetinib with anti-PD-1 Pembrolizumab, was terminated prematurely due to lack insufficient evidence of clinical efficacy (166). Concerning Carlumab (anti-CCL2), although preliminary antitumor activity was demonstrated in different solid cancers, including NSCLC (155), it failed to prove clinical benefit in a phase II study in metastatic prostate cancer (169). Interestingly, IL-4Rα blocking antibody dupilumab given in conjunction with PD-(L)1 ICB in NSCLC patients who had progressed on ICB alone reduced circulating Mo, expanded tumor-infiltrating CD8^+^ T cells, and in one out of six patients, drove a near-complete clinical response two months after treatment, advocating for its further clinical evaluation (143).

## 6 Conclusion and future perspectives

TIMs within the TME stand as prominent culprits in fostering resistance against ICB, while the BMN serves as the principal nurturing ground for myeloid cells throughout adulthood. Based on recent and, whenever possible, scRNA-seq data, we provide a comprehensive overview of solid cancer-mediated hematopoietic alterations with particular focus on myelopoiesis in the BMN of NSCLC patients. Overall numerous hematopoiesis perturbing cues have been identified that boil down to emergency myelopoiesis following rise in TIMs, at the expense of megakaryocyte/erythroid and lymphoid differentiation. Despite the wealth of novel information provided by cutting edge technologies such as scRNA-seq, this has led to an ongoing evolution in our understanding of hematopoiesis and the identification of lung TIM subcluster, often hampering consensus in terms of their predictive value for OS to ICB. Recent studies also reveal a previously undescribed role for the MSCs in the BMN, advocating for more research into the latter instead of blood- and tumor-derived HSPCs and immune cells. Overall, these findings emphasize that NSCLC should be considered as a systemic disease, underscoring the need for further investment in biomarker monitoring by integration of innovative technologies and models that extend beyond snapshots of the primary disease site. Additionally, we believe hematopoietic perturbing cues hold untapped target potential to improve the current sobering response rates to immunotherapy for advanced NSCLC patients.

## Author contributions

EC-E: Investigation, Writing – original draft, Writing – review & editing, Visualization. KR: Writing – original draft, Investigation. TB: Writing – original draft, Investigation. YJ: Writing – review & editing. DV: Writing – review & editing. RH: Writing – review & editing. AB: Writing – review & editing. IR: Writing – review & editing. LD: Writing – review & editing. CG: Conceptualization, Funding acquisition, Investigation, Supervision, Writing – original draft, Writing – review & editing.

## Supplementary material

Supplementary Table 1 Surface markers of murine hematopoietic cell subsets.

## Glossary

**Table:**

ATF3	Activating transcription factor 3
ATP	Adenosin triphosphate
BC	Breast carcinoma
BMN	Bone marrow niche
CAR	CXCL12-abundant reticular cells
(c)DC1/2A/2B/3	(Conventional) dendritic cell type 1/2A/2B/3
CDP	Common DC Progenitors
CLP	Common Lymphoid Progenitors
cMoP	Common Mo Progenitors
CMP	Common Myeloid Progenitors
CTLA-4	Cytotoxic T-Lymphocyte Associated protein-4
CXCR2	C-X-C Motif Chemokine Receptor 2
EoMP	Eosinophil/basophil/Mast cell-progenitors
G0	Granulocyte progenitors
G1	Pro neutrophils
G2	PreNeu
G3	Immature Neu
G4, G5a,b,c	Mature Neu
G-CSF	Granulocyte colony-stimulating factor
GFs	Growth factors
GM-CSF	Granulocyte macrophage colony stimulating factor
GMP	Granulocyte/Mo Progenitors
GPs	Granulocyte Progenitors
HSC	Hematopoietic stem cells
HTRA1	HtrA serine peptidase 1
ICB	Immune checkpoint blockade
ICI	Immune checkpoint inhibitors (all replaced by ICB)
IFNγ	Interferon gamma
IGF1	Insulin growth factor 1
Iv	Intravenous
IRF8	Interferon regulatory factor 8
JAG1	Notch ligand jagged 1
KP	Mice bearing orthotopic KrasG12DTp53−/-
LCAM	Lung cancer activated module
LKs	Lin^-^Sca-1^-^c-Kit^+^ HSCs
LLC	Lewis lung carcinoma
LMPP	Lymphoid-primed Multipotent Progenitor
LOX	Lysyl oxidase
LPP	Lymphoid-Primed Progenitors
LSK	Lin^−^Sca-1^+^c-Kit^+^
LT-HSCs	Long term hematopoietic stem cells
LUAD	Adenocarcinoma
Mac	Macrophages
McDPs	Mo/cDC Progenitor
M-CSF	Macrophage colony-stimulating factor
MDPs	Mo/DC Progenitors
MDSC	Myeloid-derived-suppressor cells
MegE	Megakaryocyte/Erythroid lineage
MEPs	Megakaryocyte/Erythrocyte Progenitors
MES	Mac-enriched subtype
Mo(s)	Monocyte(s)
MoDCs	Monocyte-derived DCs
MPP 1-6	Multipotent progenitors 1-6
mregDCs	Mature regulatory DCs
MSC	Mesenchymal stromal cells
NANs	Normal adjacent tissue-associated neutrophils
NES	Neutrophils - enriched subtype
Neu	Neutrophils
NSCLC	Non-small cell lung cancer
OPs	Osteoprogenitors
OS	Overall survival
Ot	Orthotopic
PD-(L)1	Programmed Death - (Ligand) 1
PDAC	Pancreatic ductal adenocarcinoma
pDCPs	pDC progenitors
pDCs	Plasmacytoid DCs
PFS	Progression free survival
PIGF	Placental growth factor
PTHrP	Parathyroid hormone-related protein
ROS	Reactive oxygen species
sc	Subcutaneous
SCF	Stem cell factor
scRNA-seq	Single cell RNA sequencing
SDF-1 /CXCL12	Stromal-cell-derived factor 1
sRAGE	Soluble form of the receptor for advanced glycation end products
ST-HSCs	Short term hematopoietic stem cells
TAMs	Tumor-associated macrophages
TANs	Tumor-associated neutrophils
TCGA	The Cancer Genome Atlas
TFs	Transcription factors
TGFβ/β1	Transforming growth factor β/ β1
TIMs	Tumor infiltrating myeloid cells
TMB	Tumor mutational burden
TME	Tumor micro-environment
TNFα	Tumor necrosis factor alpha
VEGF (R)	Vascular endothelial growth factor (receptor)
