# Supplementary material for: The crosstalk between lung cancer and the bone marrow niche fuels emergency myelopoiesis
Source: Front Immunol. 2024 Aug 1;15:1397469. doi: 10.3389/fimmu.2024.1397469 (PMC11324509; doi:10.3389/fimmu.2024.1397469)
Supplement: Supplementary Table 1 — Surface markers of murine hematopoietic cell subsets. [file Table_1.docx]

Supplementary Table S1. Surface markers of murine hematopoietic cell subsets.

| **Cell subset** | **Surface markers** |
| --- | --- |
| HSC | LSK CD34^-^ Flt3^-^ CD48^-^ CD150^+^ |
| MPP1 | LSK CD34^+^ Flt3^-^ CD48^-^ CD150^+^ |
| MPP2 | LSK CD34^+^ Flt3^-^ CD48^+^ CD150^+^ |
| MPP3 | LSK CD34^+^ Flt3^-^ CD48^+^ CD150^-^ |
| MPP4 | LSK CD34^+^ Flt3^+^ CD48^+^ CD150^-^ |
| MPP5 | LSK CD34^+^ Flt3^-^ CD48^-^ CD150^-^ |
| MPP6 | LSK CD34^-^ Flt3^-^ CD48^-^ CD150^-^ |
| CMPs | LK CD16/32^lo^ CD34^+^ Flt3^+^ CD115^-^ |
| GMPs | LK CD16/32^hi^ CD34^+^ Flt3^-^ Ly6C^-^ |
| G0 | LK CD16/32^hi^ CD34^+^ Flt3^-^ Ly6C^+^ CD115^-^ |
| cMoPs | LK CD16/32^hi^ CD34^+^ Flt3^-^ Ly6C^+^ CD115^+^ |
| Mo | Sca-1^-^ c-Kit^-^ CD16/32^h^i CD34^-^ Flt3^-^ Ly6C^+^ CD115^+^ |
| MDPs | LK CD16/32^lo^ CD34^+^ Flt3^+^ Ly6C^-^ CD115^+^ |
| CDPs | Lin^-^ Sca-1^-^ c-Kit^lo^ CD16/32^lo^ CD34^+^ Flt3^+^ Ly6C^-^ CD115^+^ |
| cDC1 | CD45^+^ CD11c^+^ MHCII^+^ XCR1^+^ |
| cDC2 | CD45^+^ CD11c^+^ MHCII^+^ CD172a^+^ |
| pDC | CD45^+^ B220^+^ CD172a^+^ |
| Basophils | CD45^+^ CD11b^+^ FceR1^+^ |
| Eosinophils | CD45^+^ CD11b^+^ CD172a^+^ SiglecF^+^ |
| G1 | CD45^+^ c-Kit^+^ CD16/32^+^ Ly6C^+^ CD115^-^ CD81^+^ |
| G2 | CD45^+^ CD115^-^ SiglecF^-^ Gr1^+^ CD11b^+^ c-Kit^+^ CXCR4^+^ |
| G3 | CD45^+^ CD115^-^ SiglecF^-^ Gr1^+^ CD11b^+^ c-Kit^-^ CXCR4^-^ Ly6G^int^/CXCR2^-^/CD101^-^ |
| G4,G5a,b,c | CD45^+^ CD115^-^ SiglecF^-^ Gr1^+^ CD11b^+^ c-Kit^-^ CXCR4^-^ Ly6G^hi^/CXCR2^+^/CD101^+^ |
